# Measuring Responses of Dicyandiamide-, 3,4-Dimethylpyrazole Phosphate-, and Allylthiourea-Induced Nitrification Inhibition to Soil Abiotic and Biotic Factors

**DOI:** 10.3390/ijerph18137130

**Published:** 2021-07-03

**Authors:** Yu-Pin Lin, Andrianto Ansari, Lien-Chieh Cheng, Chiao-Ming Lin, Rainer-Ferdinand Wunderlich, Thanh-Ngoc-Dan Cao, Hussnain Mukhtar

**Affiliations:** 1 Department of Bioenvironmental Systems Engineering, National Taiwan University, Taipei 10617, Taiwan; yplin@ntu.edu.tw (Y.-P.L.); andrianto.ansari@mail.ugm.ac.id (A.A.); smallgodcheng@gmail.com (L.-C.C.); chiaominglin@ntu.edu.tw (C.-M.L.); rainer.wunderlich@gmail.com (R.-F.W.); 2 Department of Agronomy, Faculty of Agriculture, Universitas Gadjah Mada, Yogyakarta 55281, Indonesia; 3 Graduate Institute of Environmental Engineering, National Taiwan University, Taipei 10617, Taiwan; caothanh201@gmail.com

**Keywords:** abiotic, dicyandiamide, inhibitory effect, 3,4-dimethylpyrazole phosphate, temperature, allylthiourea

## Abstract

Nitrification inhibitors (NIs) such as dicyandiamide (DCD), 3,4-dimethylpyrazole phosphate (DMPP), and allylthiourea (AT) are commonly used to suppress ammonia oxidization at different time scales varying from a few hours to several months. Although the responses of NIs to edaphic and temperature conditions have been studied, the influence of the aforementioned factors on their inhibitory effect remains unknown. In this study, laboratory-scale experiments were conducted to assess the short-term (24 h) influence of eight abiotic and biotic factors on the inhibitory effects of DCD, DMPP, and AT across six cropped and non-cropped soils at two temperature conditions with three covariates of soil texture. Simultaneously, the dominant contributions of ammonia-oxidizing archaea (AOA) and bacteria (AOB) to potential ammonia oxidization (PAO) were distinguished using the specific inhibitor 2 phenyl-4,4,5,5-tetramethylimidazoline-1-oxyl 3-oxide (PTIO). Our results revealed that AT demonstrated a considerably greater inhibitory effect (up to 94.9% for an application rate of 75 mg of NI/kg of dry soil) than DCD and DMPP. The inhibitory effect of AT was considerably affected by the relative proportions of silt, sand, and clay in the soil and total PAO. In contrast to previous studies, the inhibitory effects of all three NIs remained largely unaffected by the landcover type and temperature conditions for the incubation period of 24 h. Furthermore, the efficacy of all three tested NIs was not affected by the differential contributions of AOA and AOB to PAO. Collectively, our results suggested a limited influence of temperature on the inhibitory effects of all three NIs but a moderate dependence of AT on the soil texture and PAO. Our findings can enhance the estimation of the inhibitory effect in soil, and pure cultures targeting the AOA and AOB supported ammonia oxidization and, hence, nitrogen dynamics under NI applications.

## 1. Introduction

Ammonia-oxidizing bacteria (AOB) and ammonia-oxidizing archaea (AOA) majorly dominate ecosystem functioning by modulating the rate of nitrification in terrestrial and aquatic ecosystems [1,2,3,4]. Ammonia oxidation is also carried out by complete ammonia oxidizers (comammox) [5,6]. Soil nitrification has been observed at temperatures as low as 2 °C [7], whereas AOA isolated from croplands, non-croplands, and geothermal sources mostly exhibited temperature optima of >35 °C [4,8,9,10]. Although nitrification is an essential process of N dynamics in the ecosystem, it results in N fertilizer loss and environmental pollution in agricultural ecosystems through nitrate leaching and nitrous oxide (N_2_O) and nitric oxide (NO) emissions from the oxidation of intermediates of the nitrification process—that is, hydroxylamine (NH_2_OH) [11,12,13]. Therefore, the inhibition of nitrification, especially the first step of nitrification (ammonia oxidation), to estimate the N loss and GHG emissions is a critical topic of research. 

Several nitrification inhibitors (NIs) have been introduced to suppress nitrification [14,15,16,17]. Nitrification inhibitors can be categorized into two broad groups: (i) ammonia oxidation inhibitors—a group of NIs that inhibit the first and rate-limiting step of nitrification by targeting the ammonia monooxygenase action and chelating the copper in the active site [12]—and (ii) nitrite oxidation inhibitors—NIs groups that inhibit the $NO_{2}^{-}$ oxidation to nitrate ($NO_{3}^{-}$). Among the NIs, dicyandiamide (DCD), 3,4-dimethylpyrazole phosphate (DMPP), and allylthiourea (AT) are commonly used to suppress the nitrification process by inhibiting soil ammonia oxidization in various environmental systems, such as fertilized grasslands, cropped soils [12,18,19], and pure cultures of ammonia oxidizers [20]. Regarding the chemical characteristics, DCD is less volatile and less soluble in water than in DMPP, whereas the toxicity of DCD is higher than that of DMPP, especially for long-term applications [21]. Regarding the inhibitory effect (IE), studies have reported a considerably (ten times approximately) higher inhibitory effect (IE) of DMPP than that of DCD on various soil ecosystems [22,23]. Shen et al. (2013) reported that AT has a higher IE than that of DCD, while the effective concentration of AT was several times higher for the AOA culture [24]. Moreover, a meta-analysis on the IE of NIs revealed a similar IE of DCD and DMPP at application rates of 13.13 and 2.23 kg NI ha^−1^, respectively [16]. Nevertheless, DCD is cheaper and provides higher monetary benefits than that of DMPP [21], and it is commonly used to suppress nitrification in various soil samples. 

A comprehensive understanding of the biotic and abiotic factors that influence the effectiveness and persistence of NIs in soil could help in predicting the effectiveness and suitability of NIs in different soils. Prior studies have revealed that environmental factors such as soil texture [25], temperature [16,26], and microbial activities affect the persistence and IE of DCD and DMPP. For instance, DCD is subjected to a higher sorption and microbial assimilation [27,28] than DMPP, and the effectiveness of both NIs has been reported to drastically decrease at increased temperatures because of a high microbial activity, mineralization, and nitrification rate over time [29,30]. Additionally, the effectiveness of inhibitors is reported to vary depending on the landcover type, with the lowest IE for DCD and DMPP in paddy soils and the highest IE for grasslands [16]. However, the biotic and abiotic factors that majorly influence the IE of NIs, especially AT, in a short-term ammonia inhibition assay have been seldom investigated.

A critical factor for variability in IE could be associated with the differential response of ammonia oxidizers to Nis, because all three inhibitors are reported to mainly affect the abundance and community structure of AOB rather than AOA. Ping et al. (2013), Chen et al. (2015), Li et al. (2019), and Luchibia et al. (2020) determined that the application of DCD and DMPP in maize cultivated soils and greenhouse vegetable fields considerably reduced the growth of bacterial *amoA* gene copies, whereas no significant difference was observed for archaeal *amoA* gene copies in different inhibitor treatments. In contrast, AOB exhibited considerable shifts within different treatments of NIs compared to the control [2,31,32]. A similar trend was reported for AT with various application rates, which mainly affected the activities (i.e., growth rate and potential ammonia oxidization (PAO)) of AOB than that of AOA at lab- and field-scale studies [20,31,33]. In contrast, Guo et al. (2019) reported that the application of DCD and DMPP inhibited the abundance and altered the composition of both AOB and AOA communities in agricultural soils. These observations led us to speculate that AT, DCD, and DMPP may inhibit nitrification in soil by inhibiting the activity of different ammonia oxidizer groups in different soils. Consequently, a considerable portion of ammonia oxidization caused by either AOA or AOB, especially at different temperatures [10,34], may be unaffected by the application of NIs. Therefore, understanding how the efficacy of NIs is affected by the relative contribution of AOA and AOB to PAO is essential for select effective inhibitors.

This study investigated the IE of three application rates, namely DCD, DMPP, and AT, for short-term ammonia inhibition assays in soils from various landcover types at different temperatures (432 scenarios). The goal of this study was to identify the abiotic and biotic factors majorly influencing the IE of NIs in the soil. We hypothesized that (1) AT and DMPP exhibit a significantly higher IE than DCD, and (2) IE is strongly influenced by landcover type, soil texture, differential contributions of AOA and AOB to nitrification, and incubation temperature.

## 2. Materials and Methods

### 2.1. Soil Collection

Soil samples representing cropped (CPT, CPC, and CPM) and non-cropped soils (NPT, NPC, and NPM) were collected from six locations in the hot and mesic region in Taiwan. Regarding soil textures, the soil samples CPT, CPC, NPT, and NPC were silty-loam soils, whereas CPM and NPM were loam soils. The sampling at each site was conducted through a random walk process, a stochastic process that describes a path consisting of a succession of steps in random directions towards a neighboring node to obtain a collection of node samples. The size of the sample areas ranged from approximately 0.75 ha to 4 ha. Three to four soil samples were recovered at a depth of 0–15 cm, and a composite sample was prepared for each site. Further soil samples were sieved through mesh to obtain particle sizes of <4 mm and stored at 4 °C. The soil samples were preincubated at an ambient temperature for 24 h before the nitrification inhibition experiment.

### 2.2. Distinguishing the Activity of AOA from AOB 

AOB- and AOA-supported nitrification were distinguished using AOA-specific inhibitor 2 phenyl-4,4,5,5-tetramethylimidazoline-1-oxyl 3-oxide (PTIO), as suggested in previous studies [33,34]. A 300-μM concentration of PTIO was used to inhibit AOA, because a PTIO concentration below or above this concentration may be insufficient to inhibit the AOA activity or inhibit a portion of AOB-supported nitrification [33,34]. In addition, 23 and 37 °C were adopted as the incubation temperatures, because the optimum temperature for AOB-supported nitrification was reported to be <25 °C and the optimum temperature for AOA-supported nitrification is >35 °C [10,34]. Furthermore, soil slurry for few soil samples were treated with AOB-specific inhibitor 1-octyne (≈aqueous concentration of 4 μmol L^−1^) to validate the PTIO-based differential contribution of AOA and AOB to nitrification in preliminary experiments. 

### 2.3. Experimental Design

**Figure 1:**
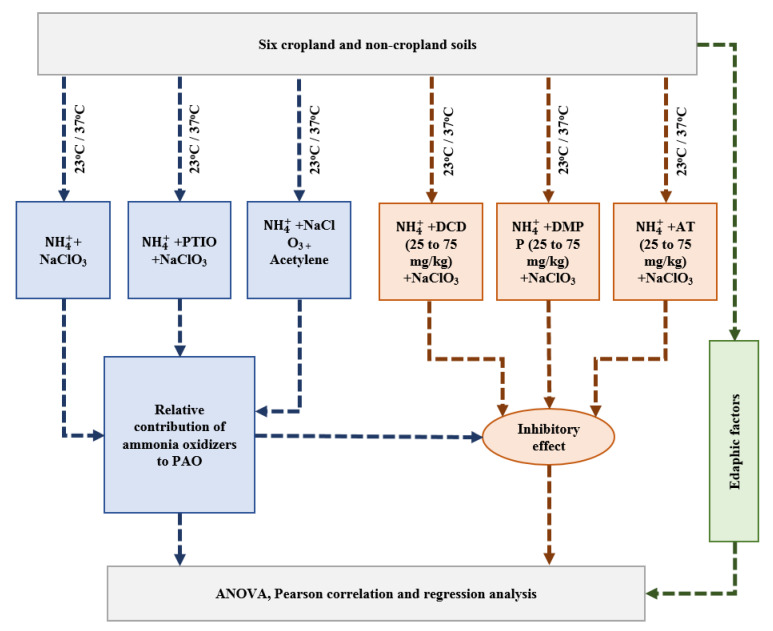
Flowchart of the estimation of short-term inhibitory effects on the soils.

Based on prior research [25,33], three application rates (25, 50, and 75 mg/kg of dry soil) for each NI (DCD, DMPP, and AT) were selected to study the effect of NIs on an ammonia inhibition assay. The field soil samples (equivalent to 5 g of dry soil weight) were mixed with 20 mL of the substrate solution containing 200 mg/L of ammonium sulfate and 1 mM of phosphate buffer (pH 7.2) and exposed to three different application rates (25, 50, and 75 mg/kg of the dry soil) of DCD, DMPP, and AT (three NIs × three application rates). Two controls (slurry in the absence of DCD, DMPP, and AT) with a specific inhibitor of AOA (that is, 300 μM of PTIO) and without a PTIO inhibitor were prepared. An additional control comprised soil suspensions, to which acetylene was added (1.0 ± 0.05 kPa) to completely inhibit the activity of AOA and AOB in the soil slurries (Figure 1). Acetylene was used to evaluate the possibility of other chemical or biological activities (e.g., heterotrophic nitrification) contributing to the $NO_{2}^{-}$ dynamics, along with ammonia oxidizers. The 10 mM of sodium chlorate (NaClO_3_) were used to inhibit the nitrite-oxidizing bacteria, as suggested in previous studies [35,36]. Two sets of treatments (including three controls) were prepared, and the soil slurries were incubated at 23 °C and 37 °C. Two replicas for each treatment were prepared to measure the initial and final $\;NO_{2}^{-}\;$ concentrations due to the small amount of soil slurry used in this experiment. The soil PAO was measured using the rate of change of the $\;NO_{2}^{-}$ concentrations over 24 h. All treatments were replicated three times. The total organic carbon (TOC), total nitrogen (TN), $NO_{2}^{-}$, moisture contents, and soil texture were measured using the Walkley–Black, Total Kjeldahl Nitrogen, UV spectrophotometer, gravimetric, and hydrometer methods, respectively [37].

### 2.4. Statistical Analysis 

The nitrite ($NO_{2}^{-}$) production over time in the NH_4_^+^ + PTIO - Acetylene treatment was considered as AOB-dominant PAO in the soil, because PTIO specifically inhibits AOA activity but not AOB at a concentration of 300 µM [33,34]. AOA-supported PAO was determined by subtracting $NO_{2}^{-}$ production in the NH_4_^+^ + PTIO and Acetylene treatment from the NH_4_^+^ treatment (Figure 1). One-way and two-way ANOVA analyses following Tukey’s post hoc test were applied to analyze whether the IE significantly differed among the NI types, incubation temperatures, landcover types, and soil textural classes. Furthermore, linear regression and correlation analyses were performed to assess the impact of soil texture, *PAO*, and the relative contributions of AOA and AOB on the IE of DCD, DMPP, and AT. All statistical analyses were performed using the base functions *aov*, *cor*, *lm*, and *TukeyHSD* in the R-language program. The *IE* was calculated by using the following formula:

$$IE\;\%=\frac{PAO_{control}-PAO_{NI}}{PAO_{control}\;}\times 100$$

## 3. Results 

### 3.1. Soil Characteristics

All soil samples were slightly acidic; the pH ranged from 6.48 to 6.71 and 6.16 to 6.26 for cropped and non-cropped soils, respectively. The moisture contents of the soil samples at the time of collection were 22.2–39.4% and 7.93–19.1%, whereas the TN levels were from 1.23–1.54 g/kg and 0.91–1.49 g/kg for cropped and non-cropped soils, respectively. The complete details of the edaphic properties of the collected samples are listed in Table 1.

### 3.2. Contribution of AOA and AOB to Soil Ammonia Oxidization 

**Figure 2:**
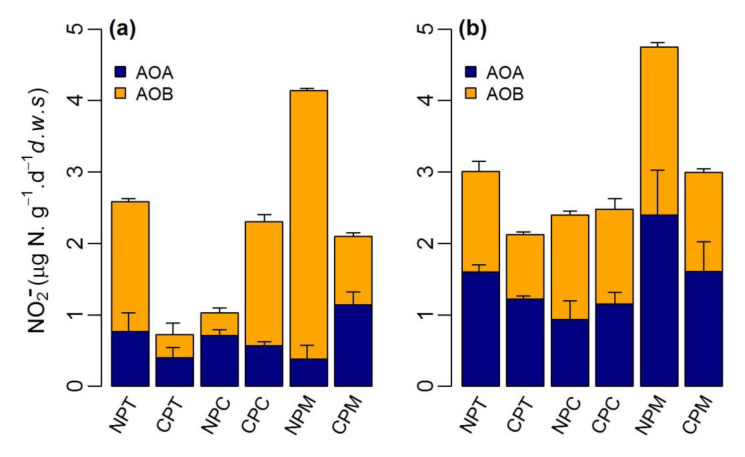
Relative contribution of ammonia-oxidizing archaea (AOA) and bacteria (AOB) to potential ammonia oxidization (PAO) at (**a**) 23 °C and (**b**) 37 °C. The first two digits of the soil name represent the landcover type: NP: non-cropland and CP: cropland, whereas the last digit refers to the soil location (see Table 1).

The PAO was significantly higher at 37 °C than at 23 °C. On average, the PAO at 23 °C was 1.71 ± 0.86 μg N·g^−1^·d^−1^ and 2.58 ± 1.56 μg N·g^−1^·d^−1^, which were 0.88 μg N·g^−1^·d^−1^ and 0.85 μg N·g^−1^·d^−1^ at 37 °C for cropped and non-cropped soils, respectively. However, no significant difference (*p* > 0.05) in the PAO was observed between cropped and non-cropped soils. Similarly, the relative contribution of ammonia oxidizers to the PAO varied more strongly with temperature than with landcover. Among the different incubation temperatures, the AOB-supported nitrification was 1.96 ± 1.72 μg N·g^−1^·d^−1^ and 1.0 ± 0.70 μg N·g^−1^·d^−1^, which was considerably higher than the AOA-supported nitrification at 23 °C (0.62 ± 0.21 μg N·g^−1^·d^−1^ and 0.71 ± 0.39 μg N·g^−1^·d^−1^) for cropped and non-copper soils, respectively. By contrast, AOA-supported nitrification was considerably higher among all the soils at 37 °C (1.64 ± 0.73 μg N·g^−1^·d^−1^ and 1.33 ± 0.24 μg N·g^−1^·d^−1^ for non-cropped and cropped soils, respectively) than at 23 °C. Nevertheless, the contribution of both AOA and AOB to PAO was not significantly different at 37 °C (Figure 2).

### 3.3. Effect of NI Type and Application Rates on the Inhibition of Ammonia Oxidization 

To compare the IE among the NIs, the data were characterized according to NI type and their application rates. On average, the largest inhibitor effect was for AT (60.0% ± 17.5%, 75.0% ± 11.3%, and 83.0% ± 7.2%), followed by DMPP (30.8% ± 22.9%, 39.9% ± 21.8%, and 47.7% ± 22.2%) and DCD (21.1% ± 17.1%, 35.9% ± 16.9%, and 44.9 ± 14.9%) at the three application rates of 25, 50, and 75 mg of NI/kg of dry soil, respectively. Moreover, the IEs of all three NIs considerably increased with the increase in the NI application rates. The difference in IE was more obvious between 50 mg of NI/kg of dry soil and 75 mg of NI/kg of dry soil compared with the difference in IE between 25 mg of NI/kg of dry soil and 50 mg of NI/kg of dry soil, especially for DCD and DMPP. However, no significant difference was observed in the IE between DCD and DMPP, especially at low application rates.

### 3.4. Effect of Soil Abiotic and Biotic Factors on the Inhibitory Effect

#### 3.4.1. Incubation Temperature 

We tested the extent to which the IE varied among the incubation temperatures in the short-term ammonia inhibition assay. Table 2 displays the ANOVA analysis of the IE of DCD, DMPP, and AT at 23 and 37 °C. Although the IE of DCD, DMPP, and AT at a given application rate varied between 23 and 37 °C, no significant difference (*p* > 0.05) was observed among the incubation temperatures. This observation was true for all three inhibitors, regardless of the application rate (Table 2). The IE of DCD, DMPP, and AT varied from 7.5% to 68.0%, 19.1% to 71.2%, and 45.3% to 84.7% at 23 °C, respectively, for cropped soils. By comparison, the IEs were 11.02–65.9%, 15.2–74.3%, and 41.9.0–90.5%, respectively, at 37 °C among all the application rates.

#### 3.4.2. Landcover Type and Soil Texture 

**Figure 3:**
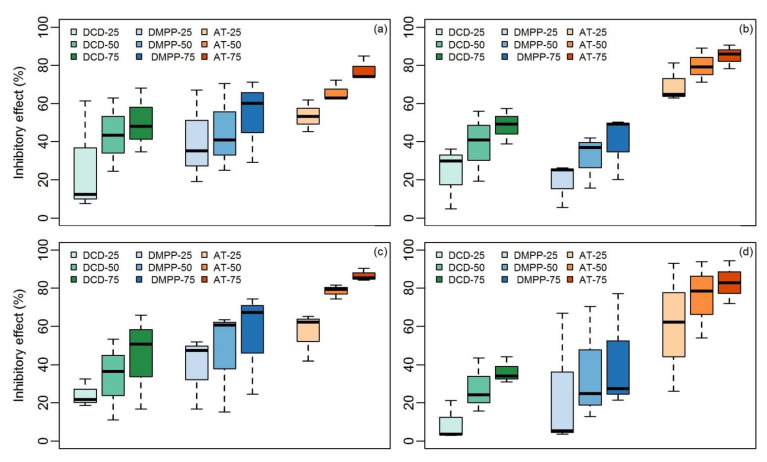
Boxplot of the inhibitory effect (IE) in non-croplands and croplands treated with dicyandiamide (DCD), 3,4-dimethylpyrazole phosphate (DMPP), and allylthiourea (AT) at three application rates (25 mg of NI/kg of dry soil, 50 mg of NI/kg of dry soil, and 75 mg of NI/kg of dry soil) incubated at two temperatures. (**a**) croplands, 23 °C; (**b**) non-croplands, 23 °C; (**c**) croplands, 37 °C; and (**d**) non-croplands, 37 °C.

The IE of DCD, DMPP, and AT did not significantly vary between cropped and non-cropped soils (Table 2, Figure 3). The IEs of DCD at the three application rates of 25, 50, and 75 mg of NI/kg of dry soil in croplands were 27.1% ± 29.8%, 43.6% ± 19.2%, and 50.2% ± 16.8%, respectively, at 23 °C and 24.4% ± 7.2%, 33.6% ± 21.2%, and 44.4% ± 25.2%, respectively, at 37 °C (Figure 3). Similarly, the IEs of DCD at the three different application rates of 25, 50, and 75 mg of NI/kg of dry soil in non-croplands were 23.6% ± 16.6%, 38.7% ± 18.4%, and 48.5% ± 9.3%, respectively, at 23 °C, whereas those at 37 °C in non-croplands were 9.2% ± 10.4%, 27.8% ± 14.2%, and 36.3% ± 6.9%, respectively (Figure 3). A similar trend was observed for DMPP and AT but with no significant difference (*p* > 0.05) between croplands and non-croplands, regardless of the incubation temperature. Moreover, the textural classes, classified on the basis of the relative proportions of silt, sand, and clay, followed a similar pattern; the IE of DCD and DMPP did not vary significantly (*p* > 0.05) among the textural classes, regardless of the application rates.

**Figure 4:**
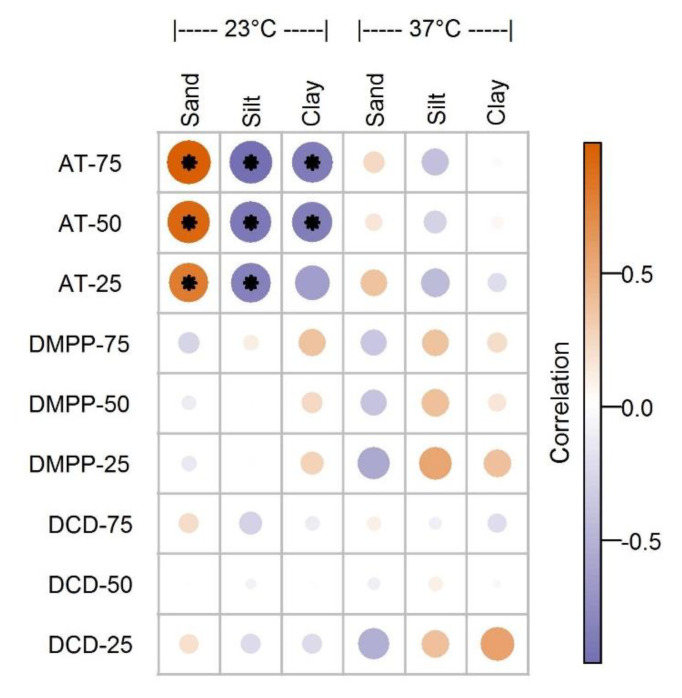
Pearson correlation between the inhibitory effects of dicyandiamide (DCD), 3,4-Dimethylpyrazole Phosphate (DMPP), and allylthiourea (AT) and the soil texture at 23 and 37 °C. Circle diameter and color represent the magnitude of the correlation. * Indicates statistical significance (*p* < 0.05).

We tested the extent to which the relative proportions of sand, silt, and clay influenced the IEs of DCD, DMPP, and AT. Figure 4 illustrates the Pearson correlation between the IEs of all three NIs and the soil texture at 23 and 37 °C. At 23 °C, the IE of AT significantly increased with the relative proportion of sand in the soil, whereas the opposite trend was observed for the relative proportions of silt and clay. However, we found no significant correlations at 37 °C. Moreover, the IEs of both DCD and DMPP exhibited weak and nonsignificant correlations with the soil texture at 23 and 37 °C, regardless of their application rates.

#### 3.4.3. Potential Ammonia Oxidation 

**Figure 5:**
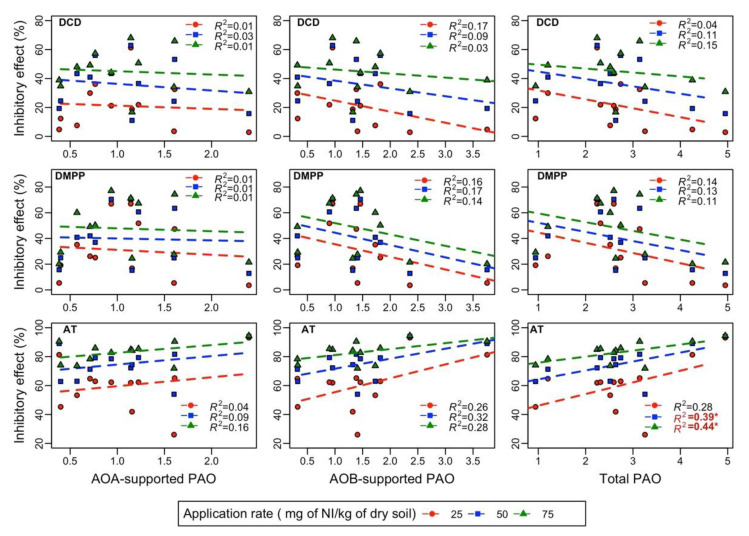
Relationship between the inhibitory effect (%) and ammonia-oxidizing archaea (AOA)-supported potential ammonia oxidization (PAO), ammonia-oxidizing bacteria (AOB)-supported PAO, and total PAO obtained using linear regression. * *p* < 0.05.

The effects of AOA- and AOB-supported PAO and total PAO on the IE of DCD, DMPP, and AT were investigated using a linear regression analysis (Figure 5). The IE of AT considerably increased with an increase in PAO (*R^2^*: 0.39 and 0.44 for an application rate of 50 and 75 mg of NI/kg of dry soil, respectively, *p* < 0.05) in the soil, except at a low application rate of 25 mg of NI/kg of dry soil. Although the IE of DCD and DMPP was negatively correlated with PAO (Figure 5), the correlation was weak and nonsignificant (*p* < 0.05). Moreover, the IE of all three NIs revealed a weak and nonsignificant correlation with the AOA- and AOB-supported PAO. 

## 4. Discussion

The investigation of the effects of abiotic and biotic factors on the effectiveness of NIs is a research topic of major interest. NIs have been used to suppress the activity of ammonia oxidizers and, hence, nitrification and nitrous oxide emissions from terrestrial and agricultural ecosystems. Under long-term incubation conditions (several days to months), the persistence and IE depend on the type of NI applied [16], incubation temperature [26,30], and edaphic conditions [16]. However, the dependence of the IE of NIs on the aforementioned factors may differ over short periods (24 h) of time, because the sensitivity of NIs to edaphic conditions and the temperature is likely to be time-dependent [16,26,38]. By applying three NIs targeting AOA- and AOB-dominated PAO, we demonstrated that (1) the IE of AT surpassed that of DCD and DMPP; (2) DCD and DMPP exhibited comparable IEs; and (3) the IEs of all three NIs revealed a weak response to the differential contribution of AOA and AOB to PAO, edaphic conditions (especially for DCD and DMPP), and temperature in the short-term ammonia inhibition assay.

As predicted, our study indicated that the IE of AT was considerably higher than that of DCD and DMPP. The literature on the comparison of the IEs of AT, DCD, and DMPP for a short-term ammonia inhibitory assay is limited, and the effectiveness of these inhibitors has only been examined for a handful of soils and pure cultures [39,40]. Li et al. (2020) reported a relatively long persistence of ammonium in soil treated with DMPP, but there was no significant difference between AT and DCD in terms of ammonia oxidization. Furthermore, Subbarao et al. (2008) demonstrated that the amount of DCD required to achieve 80% inhibition (ED_80_) was several orders of magnitude higher than that of AT [40]. Our results, however, do not corroborate either result but, instead, suggest a significant difference, although less extreme (<50%), between the IEs of DCD and AT. Similarly, we could not corroborate the higher IE of DMPP (approximately ten times) compared with DCD, as reported in previous studies [22,23]. The IE of DMPP was almost identical to that of DCD. This discrepancy could be partially explained by the duration of the ammonia inhibitory assays. This is because the IE of NIs depends on degradation—that is, sorption, mineralization, and microbial assimilation over time [16,26,38]. Since the degradation of NIs is limited in short-term incubation experiments, their IEs are more likely to be similar. However, the dose-response functions and, therefore, the quantity of DCD or DMPP required to achieve the same IE as exhibited by AT remain unknown.

Initially, we expected the temperature to strongly affect the IE of all three NIs. This expectation was based on the reported modulation of NI persistence, PAO, the relative contribution of AOA and AOB to PAO, and microbial assimilation by temperature [29,30]. In contrast to our hypothesis and prior research, the IEs of all three NIs were largely unaffected by temperature. This discrepancy could be attributed to (1) the limited impact of microbial assimilation on NIs for short-term incubation experiments and (2) the weak correlations between IE and AOA and AOB associated PAO and the total PAO (except AT). Although the IE of AT was moderately correlated with PAO, the IE was not significantly dependent on temperature, despite the observed PAO–temperature relationship (Figure 5). This is, because the variation in PAO among the soils was influenced by both the soil type and temperature. However, it is important to mention that the differential contributions of AOA and AOB ammonia oxidization may be affected by the sensitivity of some AOB species to the AOA-specific inhibitor PTIO [24] or the presence of a different abundance of comammox [5,6], which deserve additional research.

In contrast to the prior research that reported soil texture as the most critical parameter influencing the IE of DMPP [25], the IEs of DCD and DMPP were not dependent on the landcover type or soil texture at all the application rates. For AT, however, we observed a moderate response to the soil texture at 23 °C. The effectiveness of AT increased with the proportion of sand but decreased with the proportions of silt and clay. These contrasting trends could be explained by the high NI adsorption on the clay and OM surfaces or, possibly, limited chemical and physical interactions in the sandy soils [15,25]. However, this observed discrepancy for DCD and DMPP (except for AT) may be attributed to the limited variations in the soil textures in our study or, potentially, the antagonistic effects between the edaphic factors influencing the IEs of DCD and DMPP. More research targeting a wider range of AOA and AOB inhibitors, soil textures, and additional microbial taxa (including *Comammox*) is encouraged. 

## 5. Conclusions

All three NIs exhibited the potential to inhibit both AOA- and AOB-supported nitrification. Notably, the IEs of DCD, DMPP, and AT were largely independent of the edaphic conditions and temperatures over the short-term incubation period (24 h). At 23 °C, however, the IE of AT was dependent on the soil texture and, to some extent, on the PAO. These results differed from those of prior studies, which focused on the effectiveness of NIs in suppressing ammonia oxidization in various types of soils for long-term ammonia inhibition. Thus, a greater consideration should be given to the interaction between the NI effectiveness and abiotic and biotic factors and their dependence on the duration of the inhibitory assay. Our results can also serve as a (1) guideline for selecting an effective inhibitor and an appropriate application rate to suppress ammonia oxidation fully or partially in soils at the laboratory and field scales and (2) enhance the understanding of ammonia prolongation in fertilized soils and nitrogen dynamics in terrestrial and aquatic ecosystem. 

## Figures and Tables

**Table 1:** Edaphic properties of the selected cropped and non-cropped soil samples (mean values).

Type	Abbreviation	pH	TN (g/kg)	TOC (g/kg)	C/N Ratio	Moisture Contents (%)	Sand (%) ^a^	Silt (%) ^a^	Clay (%) ^a^	Textural Classes ^b^
Cropped soil	CPT	6.71	1.23	9.94	8.14	22.2	19.1	61.4	19.5	Silty loam
	CPC	6.61	1.54	12.63	8.20	33.7	17.1	59.4	23.5	Silty loam
	CPM	6.48	1.52	10.14	6.69	39.4	32.1	49.0	18.2	Loam
Non-cropped soils	NPT	6.16	0.91	8.32	9.13	17.7	33.3	51.8	14.9	Silty loam
	NPC	6.26	1.49	12.01	8.05	7.93	20.5	59.6	19.9	Silty loam
	NPM	6.24	1.14	7.91	6.99	19.1	40.8	44.0	15.2	Loam

TN: total nitrogen, TOC: total organic carbon, and C/N: organic carbon to nitrogen ratio. ^a^ Soil texture was measured using the hydrometer method. ^b^ USDA textural classes of soils.

**Table 2:** F-values from the ANOVA of the inhibitory effects.

Inhibitor Type	DCD			DMPP			AT		
Application Rates (mg/kg of Dry Soil)	25	50	75	25	50	75	25	50	75
T ^a^	0.73	1.17	1.10	0.03	0.04	0.02	0.087	0.36	0.74
T × LCT ^b^	0.31	0.01	0.11	0.01	0.02	0.01	0.30	1.60	1.69
T × C ^b^	0.20	0.06	0.21	0.83	0.31	0.01	0.64	0.08	0.01

Notes: ^a^ one-way and ^b^ two-way. T: incubation temperature, LCT: landcover type, and C: textural classes; no differences were significant (*p* > 0.05).

## Data Availability

All data used in this study can be found in figures and tables.
